# North-South Differentiation and a Region of High Diversity in European Wolves (*Canis lupus*)

**DOI:** 10.1371/journal.pone.0076454

**Published:** 2013-10-11

**Authors:** Astrid V. Stronen, Bogumiła Jędrzejewska, Cino Pertoldi, Ditte Demontis, Ettore Randi, Magdalena Niedziałkowska, Małgorzata Pilot, Vadim E. Sidorovich, Ihor Dykyy, Josip Kusak, Elena Tsingarska, Ilpo Kojola, Alexandros A. Karamanlidis, Aivars Ornicans, Vladimir A. Lobkov, Vitalii Dumenko, Sylwia D. Czarnomska

**Affiliations:** 1 Mammal Research Institute, Polish Academy of Sciences, Białowieża, Poland; 2 Department of Biosciences, Aarhus University, Aarhus, Denmark; 3 Aalborg University, Department 18/Section of Environmental Engineering, Aalborg, Denmark; 4 Aalborg Zoo, Aalborg, Denmark; 5 Department of Biomedicine, Aarhus University, Aarhus, Denmark; 6 Laboratorio di Genetica, Istituto Superiore per la Protezione e la Ricerca Ambientale, Ozzano Emilia (BO), Italy; 7 Museum and Institute of Zoology, Polish Academy of Sciences, Warszawa, Poland; 8 Institute of Zoology, National Academy of Sciences of Belarus, Minsk, Belarus; 9 Department of Zoology, Biological Faculty, Ivan Franko National University of Lviv, Lviv, Ukraine; 10 Department of Biology, Faculty of Veterinary Medicine, University of Zagreb, Zagreb, Croatia; 11 BALKANI Wildlife Society, Sofia, Bulgaria; 12 Finnish Game and Fisheries Research Institute, Oulu, Finland; 13 ARCTUROS, Civil Society for the Protection and Management of Wildlife and the Natural Environment, Thessaloniki, Greece; 14 Department of Ecology and Natural Resources Management, Norwegian University of Life Sciences, Ås, Norway; 15 Latvian State Forest Research Institute “Silava”, Salaspils, Latvia; 16 Zoological museum of Odessa, National I.I. Mechnikov University, Odessa, Ukraine; 17 Biosphere Reserve Askania Nova, Askania-Nova, Chaplynka District, Kherson Region, Ukraine; University of York, United Kingdom

## Abstract

European wolves (*Canis lupus*) show population genetic structure in the absence of geographic barriers, and across relatively short distances for this highly mobile species. Additional information on the location of and divergence between population clusters is required, particularly because wolves are currently recolonizing parts of Europe. We evaluated genetic structure in 177 wolves from 11 countries using over 67K single nucleotide polymorphism (SNP) loci. The results supported previous findings of an isolated Italian population with lower genetic diversity than that observed across other areas of Europe. Wolves from the remaining countries were primarily structured in a north-south axis, with Croatia, Bulgaria, and Greece (Dinaric-Balkan) differentiated from northcentral wolves that included individuals from Finland, Latvia, Belarus, Poland and Russia. Carpathian Mountain wolves in central Europe had genotypes intermediate between those identified in northcentral Europe and the Dinaric-Balkan cluster. Overall, individual genotypes from northcentral Europe suggested high levels of admixture. We observed high diversity within Belarus, with wolves from western and northern Belarus representing the two most differentiated groups within northcentral Europe. Our results support the presence of at least three major clusters (Italy, Carpathians, Dinaric-Balkan) in southern and central Europe. Individuals from Croatia also appeared differentiated from wolves in Greece and Bulgaria. Expansion from glacial refugia, adaptation to local environments, and human-related factors such as landscape fragmentation and frequent killing of wolves in some areas may have contributed to the observed patterns. Our findings can help inform conservation management of these apex predators and the ecosystems of which they are part.

## Introduction

Population genetic structure can occur across relatively short distances in the absence of geographic barriers in highly mobile species, such as lynx (*Lynx canadensis*) [1], coyotes (*Canis latrans*) [2], and wolves (*C. lupus*) [3]. Wolves are now recolonizing several areas of Europe, including western Poland and eastern Germany, France, and Switzerland (e.g. [4], [5]). Colonization processes are still poorly understood and, despite legal protection in most European countries, illegal killing and accidental mortality remain widespread threats to wolf survival [6], [7]. Previous studies using mitochondrial DNA (mtDNA) and microsatellite markers suggested a highly divergent Italian population with relatively low genetic diversity following long-term isolation and an extensive bottleneck [8], [9]. More information on European wolf population structure and the location of and divergence between population clusters is needed to understand evolutionary history and inform conservation management.

Previous findings based on 14 microsatellite loci [3] suggested that southern and northcentral European wolves may comprise one population, bisected by a second population extending from eastern Poland into Belarus, Ukraine, and Russia. It was nevertheless noted that these clusters may comprise further substructure, because of the additional clusters indicated by the mtDNA results. Although southern and northcentral European wolves were grouped into one population, four individuals from southern Europe (Greece and Bulgaria) included in an analysis of 48K single nucleotide polymorphism (SNP) markers appeared to be divergent from wolves in northcentral European countries including Poland and Lithuania [10]. Moreover, recent investigations of morphology [11] and ecology [12] in various parts of Europe suggest that previously defined population clusters might be further resolved.

Earlier analyses typically examined <50 markers, whereas new genomic tools such as SNP markers permit typing of several thousand loci and, with an adequate sample of representative individuals, improved resolution of population genetic structure and evolutionary processes (e.g. [13]). Microsatellites typically have rapid mutation rates, and a bias toward highly polymorphic loci might result in overestimates of genetic diversity [14]. Consequently, amplification of even a few hundred SNPs should improve evaluation of genetic profiles compared with a smaller panel of microsatellite markers. We examined spatial genetic patterns in European wolves to determine whether results based on SNP analyses 1) appear consistent with previous findings from mtDNA and microsatellites, and 2) improve resolution of population genetic structure across the continent. Although our study focused on wolves, the results may help understand patterns of genetic variation, population structure, and gene flow in other highly mobile species that occur at low densities.

## Materials and Methods

### DNA Extraction and Genotyping

All samples were collected from animals found dead or from wolves legally harvested for purposes other than research. The project was carried out under contract (no. 4184/B/P01/2009/37) with the Polish Ministry of Science and Higher Education in compliance with all requirements. No ethics permit was required as the project did not involve collection of samples from live animals. Samples from Finland, Latvia, Russia, Belarus, Ukraine, Slovakia, Croatia, Bulgaria, and Greece were obtained from collaborators and used with their permission.

We extracted genomic DNA from tissues of n = 272 European canids sampled 1995–2010, using a DNeasy Tissue Kit (Qiagen) according to the manufacturer’s protocol. We performed DNA quantity and purity control using the spectrophotometer NanoDrop ND-1000 (NanoDrop Technologies, Inc., Wilmington, Delaware, USA) and examined DNA quality using electrophoresis with a 1% agarose gel. Samples were genotyped at AROS Applied Biotechnology A/S in Aarhus, Denmark, for 170 000 loci using the CanineHD BeadChip microarray from Illumina® (Illumina, Inc., San Diego, California, USA) according to their Infinium HD Assay Ultra assay protocol. Samples included n = 20 Italian dogs and three known first-generation captive wolf-dog hybrids to help identify and remove individuals suspected to have dog ancestry. The dogs were of unknown breed/ancestry sampled in villages close to wolf distributions. Four wolf samples (from Belarus, Greece and Ukraine) were processed and genotyped in duplicates to verify genotyping reliability and showed consistent individual profiles.

**Figure 1:**
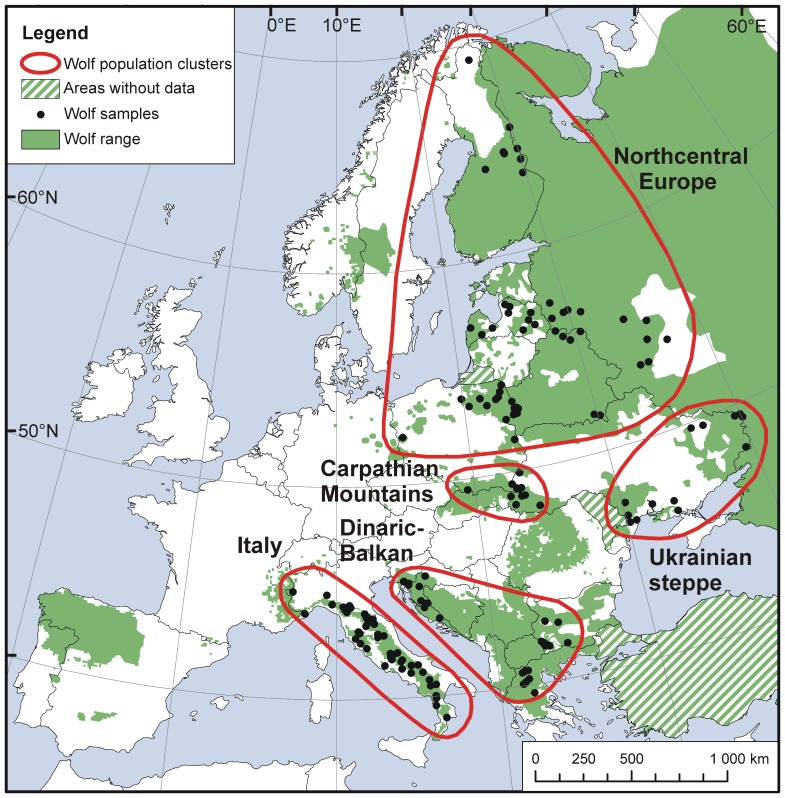
Map of European wolf distribution showing wolf samples (n = 177) and population clusters. Map of European wolf distribution showing wolf samples (n = 177) and population clusters detected using 67K single nucleotide polymorphism (SNP) markers. Distribution map prepared by the Large Carnivore Initiative for Europe (lcie.org) based on Linnell et al. 2008. Wolves also occur in areas marked ‘without data’, but their distribution is uncertain.

We used GenomeStudio™ and accompanying guidelines from Illumina [15] to call genotypes for analyses of wolf genetic structure (Table S1). Italian canids may have higher levels of relatedness due to their isolated status [8], [9]. Accordingly, we performed a separate evaluation for Italian wolves (Table S1). We determined pairs of wolves with an identity-by-descent score of >0.5 (equivalent of parent-offspring or sibling relations) using PLINK [16] and removed one individual per pair to reduce the influence of relatedness among individuals on population genetic structure. The screening resulted in a sample of n = 177 European wolves (Fig. 1). We estimated genetic variation, including observed and expected heterozygosity, and the percentages of polymorphic loci, missing alleles, and loci not in Hardy-Weinberg equilibrium (HWE) in PLINK for the Italian (n = 50) and other European wolves (n = 127) based on 79 536 autosomal SNPs prior to applying filters for genotyping and minor allele frequency. Subsequently, we performed quality control for a merged file of 79 462 SNPs for the 177 wolves (Table S2). This resulted in a final data set of 67 784 (67K) high-quality autosomal SNPs for further analyses.

### Statistical Analyses of Genetic Structure

We performed principal component analyses (PCA) on a subset of markers pruned for linkage disequilibrium in PLINK (we removed SNPs with pairwise genotypic associations (r^2^)>0.8 within a window of 50 SNPs) using the *adegenet-*package [17] in R 2.14.2 [18]. Subsequently, we evaluated population genetic structure using a Bayesian inference model in the program STRUCTURE 2.3.3 [19]. The STRUCTURE approach has become a standard method of evaluating the number of genetic clusters in the data set while assuming equilibrium genetic conditions (Hardy-Weinberg and linkage equilibrium). These conditions may nonetheless not be fulfilled in all situations, including that of wolves sampled across the European continent. Thus it could be informative to also evaluate data with PCA methods that are 1) without such equilibrium assumptions, and 2) better able at identifying transitions in genetic profiles more accurately described as clines, which may be more difficult to detect than clusters [20].

We used 10 000 burn-in runs followed by 10 000 Markov chain Monte Carlo repetitions in STRUCTURE and evaluated K = 1–10 possible population clusters. Each parameter setting was repeated three times. We used the admixture model and allowed allele frequencies to be correlated among populations. Initial assessments confirmed previous reports of an isolated Italian wolf population [8], [9], and the separation was sufficiently strong that it was necessary to remove the Italian wolves to resolve the remaining samples into biologically meaningful clusters (data not presented). We therefore divided the data set and investigated structure within Italy and the remainder of Europe separately using K = 1–10. We used STRUCTURE Harvester v.06.92 [21] and CLUMPP v1.1.2 [22] to summarize the output, which included estimates for Delta K [23], and plotted individual assignments with Distruct v1.1 [24]. We estimated the observed and expected heterozygosity, and the percentage of loci not in HWE, for the major population clusters in PLINK [16]. Finally, we calculated F_ST_ between all pairs of population clusters identified by PCA and STRUCTURE using GENEPOP v. 4.1.4 [25].

## Results

### Genetic Variation

Observed and expected heterozygosity was lower in Italian wolves than in the rest of Europe (Table 1). The percentage of missing loci was higher for the Italian population, whereas fewer loci were polymorphic. However, the Italian population showed a smaller percentage of loci not in Hardy-Weinberg equilibrium.

**Table 1:** Basic genetic measurements for data from Italian and other European wolves analysed for 79,536 autosomal single nucleotide polymorphism (SNP) markers.

Sample (sample size)	H_obs_ (SE, 95% CI)	H_exp_ (SE, 95% CI)	Percent polymorphicloci	Percent missingloci	Percent loci notin HWE†
Italy (n = 50)	0.1673 (0.0006, 0.1661–0.1685)	0.1761 (0.0006, 0.1749–0.1773)	83.79	2.201	0.35
Europe other* (n = 127)	0.2589 (0.0006, 0.2577–0.2601)	0.2800 (0.0006, 0.2788–0.2812)	99.95	0.294	0.98

Observed and expected heterozygosity (H_obs_ and H_exp_) are shown with standard error (SE) and 95% confidence intervals (CI).

* Belarus, Bulgaria, Croatia, Finland, Greece, Latvia, Poland, Russia, Slovakia, Ukraine.

† Percent loci not in Hardy-Weinberg equilibrium after Bonferroni correction for multiple tests.

### Population Genetic Structure of European Wolves

**Figure 2:**
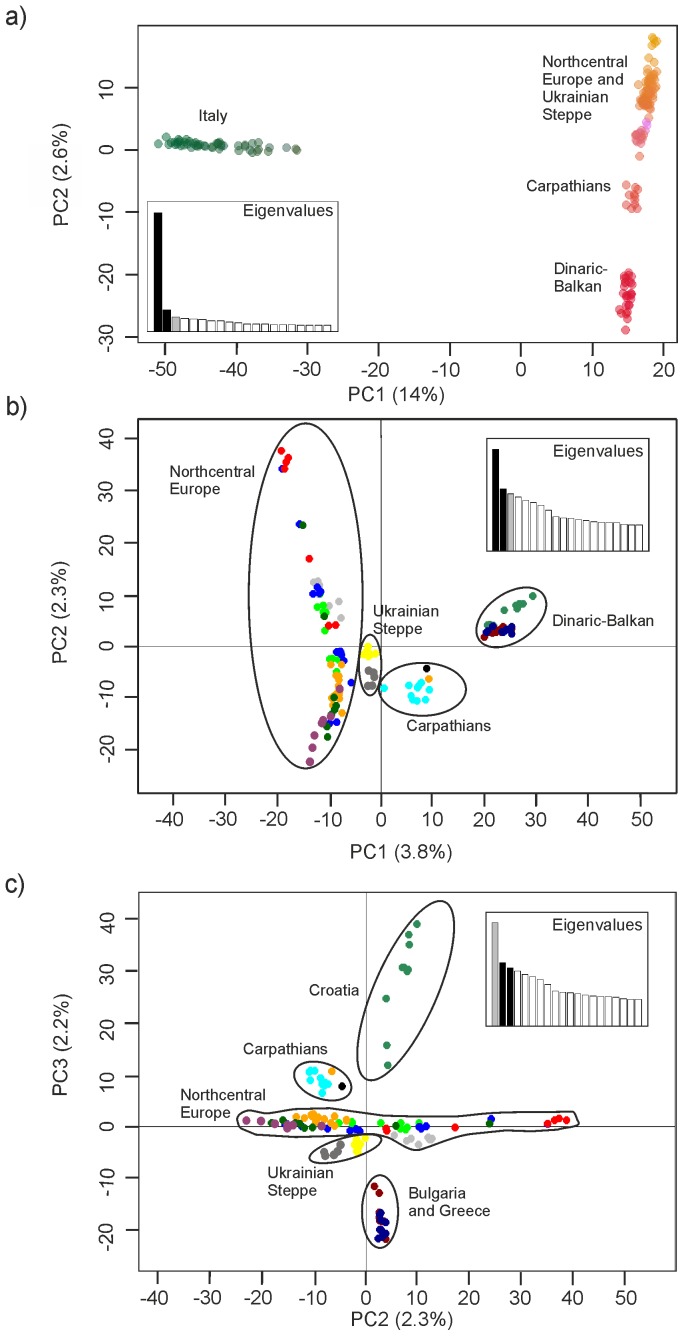
Principal component analysis of European wolves using 67K single nucleotide polymorphism markers. Principal component analysis of European wolves using 67K single nucleotide polymorphism (SNP) markers. a) Colourplot of all wolves (n = 177) where genetic similarity is represented by similar colours and spatial proximity. b) Individuals from Europe (excluding Italy and four outliers from southern Ukraine, n = 123) with the 1^st^ and 2^nd^ PC axes showing four main genetic clusters: *Dinaric-Balkan* – green: Croatia (n = 10), dark red: Bulgaria (n = 10), dark blue: Greece (n = 9); *Carpathian Mountains* – black: Slovakia (n = 1), turquoise: Western Ukraine (n = 10), orange: Polish Carpathian Mountains (n = 1); *Ukrainian Steppe* – yellow: Eastern Ukraine (n = 7), dark grey: Southern Ukraine (n = 5); *Northcentral Europe* – gray: Finland (n = 8), light green: Latvia (n = 10), blue: Russia (n = 15), red: Northern Belarus (n = 8), orange: Poland (except Polish Carpathian Mountains, n = 15), violet: Western Belarus (n = 6), dark green: Southern Belarus (n = 8). c) Individuals from Europe (excluding Italy and four outliers from southern Ukraine, n = 123) showing the 2^nd^ and 3^rd^ PC axes. Sampling and clusters as in b), except the Dinaric-Balkan cluster for which Croatia and Bulgaria/Greece formed separate groups.

The PCA revealed a highly isolated Italian population (Fig. 2a), which is visible on the first PC axis. The second PC axis reflects structuring in the rest of the European sample, and revealed no obvious differentiation within the Italian wolves. We subsequently excluded Italian wolves to resolve structuring of other European samples. Here we observed four markedly divergent individuals from southern Ukraine (Fig. S1). After removal of the four outlying southern Ukraine individuals, we found that wolves from Croatia, Greece and Bulgaria (henceforth the Dinaric-Balkan population, see [26]) formed a separate cluster (Fig. 2b). Within this cluster, Croatian wolves appear to constitute a distinct group on the third PC axis (Fig. 2c). Wolves from the Carpathian Mountains in central Europe (Slovakia and western Ukraine) occupy an intermediate position on the first axis (Fig. 2b). Another cluster comprising individuals (except the four outliers that were removed) from southern and eastern Ukraine (hereafter the Ukraine Steppe) is intermediate between Carpathian and northern European wolves (Fig. 2b).

**Figure 3:**
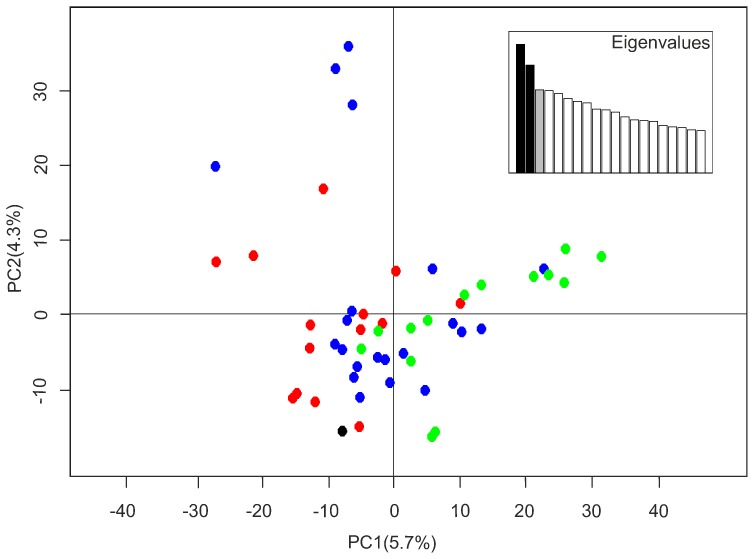
Principal component analysis of Italian wolves (n = 50) using 67K single nucleotide polymorphism markers. Principal component analysis of Italian wolves (n = 50) using 67K single nucleotide polymorphism (SNP) markers. Red = Northern Apennines (n = 14), blue = Central Apennines (n = 21), green = Southern Apennines (n = 14), black = a single sample from the Alps.

A gradient in genetic profiles within northcentral Europe is visible on the second axis, but in contrast to the first PC axis this gradient within northcentral Europe does not appear to correspond with geographic distance (Fig. 2b,c). The highest and lowest values reflect wolves from northern Belarus (and one Russian individual sampled near the border of Belarus and Latvia), and western Belarus, respectively. The variation in profiles within Belarus therefore exceeds that of all other wolves within northcentral Europe, including individuals from the northernmost sampling region of Finland. The remainder of the samples from Russia, Latvia, Poland, and southern Belarus showed high overlap among wolf profiles (Fig. 2b,c). The results for Italy (Fig. 3, Fig. S2) identified certain outliers that had been sampled in the Northern and Central Apennines (regions 1 and 2), but suggested no obvious population clusters.

**Figure 4:**
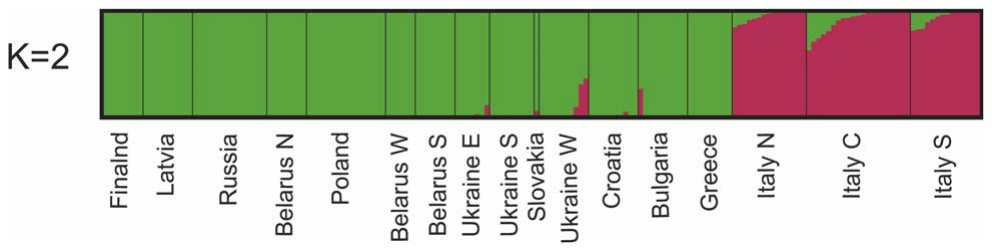
STRUCTURE results for European wolves (n = 177) using 67K single nucleotide polymorphism markers. STRUCTURE results for European wolves (n = 177) using 67K single nucleotide polymorphism (SNP) markers and K2 population clusters.

**Figure 5:**
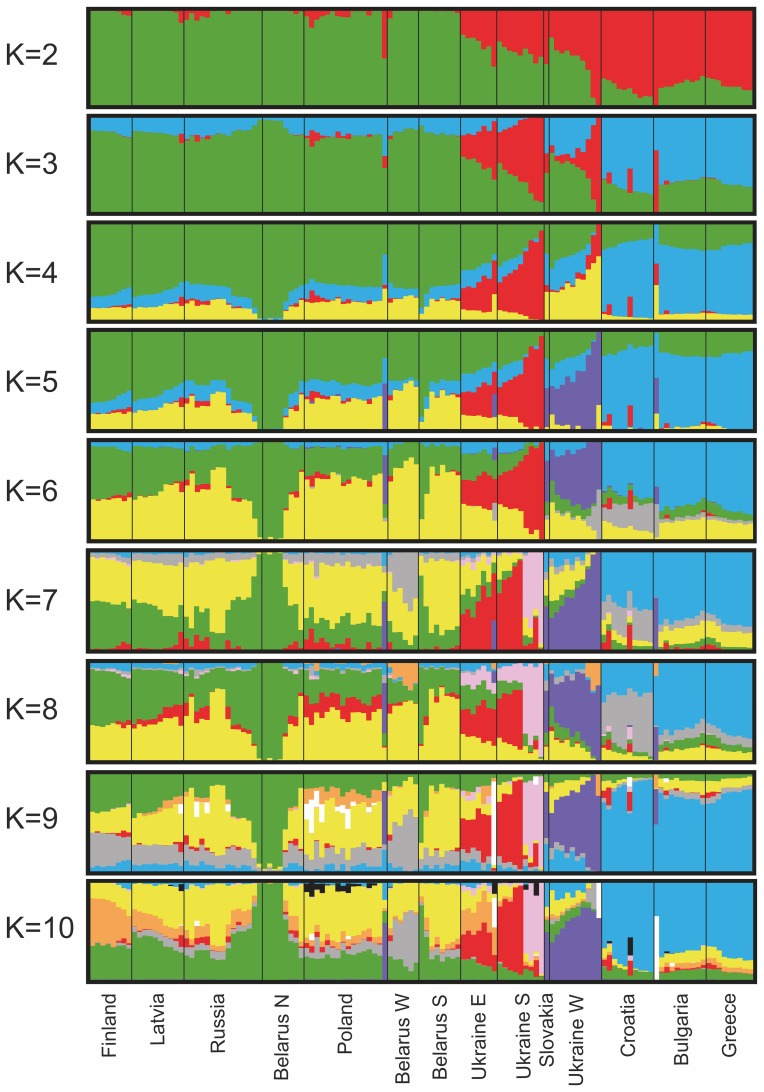
STRUCTURE results for European wolves (n = 127, without Italy and outliers). STRUCTURE results for European wolves (n = 127, without Italy and outliers) using 67K single nucleotide polymorphism (SNP) markers and K = 2–10 population clusters.

**Figure 6:**
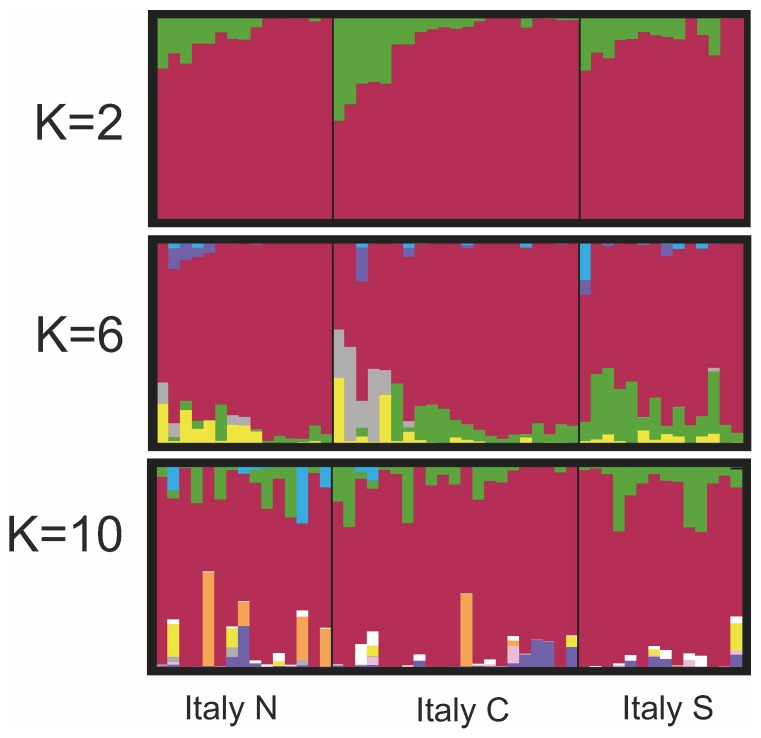
STRUCTURE results for Italian wolves (n = 50). STRUCTURE results for Italian wolves (n = 50) using 67K single nucleotide polymorphism (SNP) markers showing results for K = 2, 6, and 10. Regions within Italy are the Northern Apennines (with a single Alpine sample in the final position, n = 15), the Central Apennines (n = 21), and the Southern Apennines (n = 14).

The STRUCTURE results for all European wolves were in accord with the PCA in showing a highly divergent Italian population (Fig. 4). STRUCTURE results for European samples without Italy concurred with the PCA (Fig. 5), and there was highest support for K = 2 and subsequently K = 4 population clusters (Table S3). K = 2 showed differentiation between northcentral and southern Europe. K = 3 identified divergent profiles in Ukraine (primarily), whereas K = 4–5 suggested further differentiation between genotypes from the Carpathian Mountains and the Ukrainian Steppe. Certain individuals in northern Belarus and Russia appear to have atypical profiles (K = 4–5), whereas K = 7 identified the four southern Ukrainian outliers (Fig. S1) as a separate cluster. STRUCTURE results for Italian wolves (Fig. 6) were in accord with the findings from the PCA, and K = 2 population clusters received the highest support (Table S4). Although some individuals had divergent profiles there was no obvious geographic structure within the country.

Observed and expected heterozygosity values were markedly lower for Italian wolves than for the four other major population clusters (Table 2) and within-cluster analyses for other European wolves reduced the percentage of loci not in HWE. F_ST_ values between pairs of population clusters indicated the presence of a highly differentiated (i.e., F_ST_>0.15, [27]) wolf population in Italy (Table 3).

**Table 2:** Basic genetic measures for major population clusters of European wolves, identified by PCA and STRUCTURE analyses, based on data from 67,784

Population cluster	Sample size	H_obs_ (SE, 95% CI)	H_exp_ (SE, 95% CI)	Percent loci not in HWE†
Northcentral Europe†	60	0.2648 (0.0006, 0.2636–0.2660)	0.2744 (0.0007, 0.2730–0.2758)	0.37
Ukrainian Steppe (south andeast)	12	0.2922 (0.0007, 0.2908–0.2936)	0.2920 (0.0006, 0.2908–0.2932)	None
Carpathian Mountains (Ukrainewest, Slovakia)	12	0.2419 (0.0008, 0.2403–0.2435)	0.2505 (0.0007, 0.2491–0.2519)	None
Dinaric-Balkan (Greece, Bulgaria,Croatia)	29	0.2550 (0.0007, 0.2536–0.2564)	0.2639 (0.0007, 0.2625–0.2653)	0.22
Italy	50	0.1649 (0.0007, 0.1635–0.1663)	0.1742 (0.0007, 0.1728–0.1756)	0.39

Observed and expected heterozygosity (H_obs_ and H_exp_) are shown with standard error (SE) and 95% confidence intervals (CI).

† Finland, Latvia, Russia, Belarus (south region), and Poland. Excluding outliers from western and northern Belarus, and Russia.

**Table 3:** F_ST_ values between pairs of major European wolf population clusters identified by PCA and STRUCTURE analyses.

Cluster (n)	Northcentral Europe†(n = 60)	Ukrainian Steppe(n = 12)	Dinaric-Balkan(n = 29)	Carpathian Mountains(n = 12)
Ukrainian Steppe (n = 12)	0.030	–	–	–
Dinaric-Balkan (n = 29)	0.046	0.053	–	–
Carpathian Mountains (n = 12)	0.046	0.056	0.056	–
Italy (n = 50)	**0.197**	**0.236**	**0.218**	**0.250**

Population information is provided in Table 2. Pairwise comparisons showing high (F_ST_>0.15) differentiation are shown in bold; n – number of samples.

† Finland, Latvia, Russia, Belarus (south region), and Poland. Excluding outliers from western and northern Belarus, and Russia.

We performed additional analyses in STRUCTURE to evaluate whether outlier individuals had profiles similar to that of dogs and known wolf-dog hybrids from Italy (n = 16), including three first-generation wolf-dog hybrids. The results (data not presented) based on 21K SNPs indicated that the four outliers from southern Ukraine (Fig. S1) had dog ancestry of q_dog_≥0.10 (range 0.16–0.34). Six Italian individuals showed dog ancestry q_dog_≥0.10 (range 0.10–0.35). Three other European outliers that had been removed prior to PCA and STRUCTURE analyses showed apparent dog ancestry. These were from northern Poland (q_dog_ = 0.22), western Ukraine (q_dog_ = 0.38), and Greece (q_dog_ = 0.76). None of the wolves from Belarus showed dog ancestry.

## Discussion

Our results indicate clear genetic divergence between Italian wolves and individuals from other European countries. We found differentiation between profiles from northern and southern Europe, with individuals from the Carpathian Mountains in central Europe displaying intermediate genotypes. Our results also reveal high genetic diversity within Belarus that exceeded the variation observed in neighbouring countries.

The presence of a distinct Italian population with comparatively low values of heterozygosity accords with earlier reports of long-term isolation and relatively low genetic diversity [8], [9]. Our findings for Italy seem comparable with earlier results based on analyses of 48K SNP loci [10]. A distinct sub-population, originating from a small number of wolves dispersing from the Apennines, has also been reported in the Italian Alps [9]. We only had one sample from the Alps, and were thus unable to evaluate the existence of a separate cluster in this region. The higher percentage of missing loci for the Italian wolves may be explained by lower quality samples, as these were not fresh tissues but obtained from animals that were found dead. Reduced data quality may have augmented homozygosity values, although low variability in Italian wolves has also been reported from studies based on microsatellite [8] and mtDNA markers [28].

Our results support the presence of distinct wolf populations in Europe [3] and detected additional genetic structure. We identified one cluster in the Carpathian Mountains, which seems consistent with mtDNA and microsatellite results from Czarnomska *et al.* [29]. They found wolves from the Polish Carpathians to be divergent from individuals sampled in the northern lowlands, although the two regions lie well within wolf dispersal distance [30], [31]. Although our Carpathian samples originated from western Ukraine and Slovakia, wolves from the Polish part of this mountain range may have similar profiles.

Carpathian individuals were distinct from Dinaric-Balkan wolves, which in our sample comprised the most isolated group outside Italy according to PCA and Structure results. Importantly, however, we did not have samples from Romania, and an important research priority will be to determine whether a gradient in wolf profiles might be present and extend from the Carpathian Mountains into the Dinaric-Balkan population. Carpathian individuals were more similar to wolves from the east (i.e. the Ukrainian Steppe) than they were to wolves from northcentral Europe, although wolves in northern Poland (part of the northcentral population cluster) are nevertheless geographically closer to the Carpathian Mountains than to the Ukrainian Steppe. Factors other than geographic distance appear therefore to be important in shaping population structure. The Carpathian Mountains are a meeting point for different wolf haplogroups and subpopulations based on mtDNA analyses [3], [32]. Czarnomska *et al.* [29] noted the apparent presence of a separate cluster in eastern Poland, between the Carpathians and the northern lowlands. This accords with Pilot *et al.* [3]’s observation of a population extending from eastern Poland into southern Belarus, northern Ukraine and Russia. Gursky [33] reported a ‘wolf-free belt’ between Carpathian and lowland wolves in Ukraine, and the divergence between the Carpathian and Ukrainian Steppe cluster suggests that (effective) dispersal between these areas may be limited. Furthermore, data on morphology and population history indicate that wolves recolonized southern Ukraine from the east [34], [35]. The PCA and STRUCTURE results are generally in agreement, although PCA appears better able to identify clines and recognize clusters represented by only a few individuals. Although we used a large number of markers, departure from the expected equilibrium conditions, such as underlying genetic structure, may have affected the STRUCTURE results [19]. Similarly, F_ST_ values should be interpreted with caution considering that some clusters are based on <20 samples.

Geffen *et al*. [36] found east-west environmental gradients to be strongly associated with population structure in North American wolves, and north-south structure has also been reported (e.g. [37]). Fine-scale differentiation is documented in certain areas with abrupt environmental transitions, such as the Pacific Coast of Canada and southeastern Alaska [38], [39]. Isolation and expansion from different glacial refugia [8], [9], [40] and adaptation to local environments and ecological conditions [12], [41] may have influenced the extent and direction of gene flow in European wolves. Human-related factors such as landscape fragmentation and development [8], [42]–[44], high hunting pressure [45]–[47] including poaching [6], [7], may also have influenced patterns of dispersal. Moreover, wars and uprisings over the past 150 years seem to have exerted a strong influence on wolf dynamics in parts of the study area [48], and may thus have influenced gene flow.

Although wolves might have had an extensive distribution in northern Eurasia during the late Pleistocene [49], [50], expansion from various refugia and replacement of different lineages appear to have played an important role in structuring wolf genetic variation in Europe [32]. Subsequent admixture would nonetheless be expected to limit divergence across well-connected populations with frequent gene flow. Ecological and behavioural factors such as prey selection could therefore play a more important role than geographic distance in shaping wolf genetic structure, as reported in northcentral Europe [12], [51]. The presence and abundance of wild ungulates in Europe, with larger species generally occurring in the north, may influence the spatial organization of wolf populations in the absence of (major) barriers to dispersal. Moose (*Alces alces*) and also wild forest reindeer (*Rangifer tarandus fennicus*) are important wolf prey in areas of northern Europe [45], [47], [52] whereas southern European wolves often rely on smaller species including livestock [53], [54]. Dinaric-Balkan wolves were reported to have smaller and differently shaped skulls than individuals from the Serbian portion of the Carpathian Mountains [11]. North American research has suggested that wolf size, in particular that of males, may influence the ability to capture and handle large prey, whereas smaller wolves may be advantaged in capturing smaller and swifter species [55]. Differential selection associated with prey defence mechanisms and the traits required to overcome these (e.g. size versus speed) might influence the differentiation observed between northern and southern Europe. The possibility of natal habitat-biased dispersal, including the presence of asymmetrical dispersal between highland and lowland areas [2], [56], also merits further attention.

Wolves in Belarus exhibited unexpected diversity and structure, and western and northern Belarus wolves showed the most divergent genotypes within northcentral Europe. The country is located near the centre of our sampling area and there are no major landscape barriers to dispersal whereas wolf harvest is high [45]–[47]. We would therefore have predicted Belarus wolf genotypes to be similar to those observed in neighbouring countries. Earlier analyses identified a distinct mtDNA subpopulation in this region [3]. Although it overlapped with the sampling area for our divergent individuals, the latter did not have the haplotype (H7) typical for this (small) subpopulation, but a haplotype (H1) common throughout northeastern Europe [3]. The origin of the high diversity within Belarus is unclear, and merits further investigation. We did not observe dog ancestry in Belarussian wolves, but dogs and wolves can interbreed with golden jackals (*C. aureus*) [57] and this might have occurred in Bulgaria (A.E. Moura, unpubl. data). We were unable to evaluate this possible source of introgression, but golden jackals are not known to occur in Belarus at present [58]. The high levels of wolf harvest reported for Belarus [45]–[47] could threaten the long-term conservation of local genetic variation. Hunting mortality may have augmented immigration into Belarus, and the divergent individuals might represent long-distance migrants. Sampling of wolves farther to the east could help clarify the high diversity observed within this country.

Wolves in Finland appeared well-connected to populations in Russia and southward, despite the geographic distance. Jansson *et al*. [59] nonetheless reported signs of isolation and inbreeding in Finish wolves analysed with a set of 17 microsatellite markers, and an earlier analysis with 10 microsatellite markers suggested marked but recent differentiation among wolves in Finland and those of the Karelia and Arkhangelsk regions of Russia [60]. Such discrepancies might, at least in part, be explained by the use of different genetic markers [14], although factors such as the lower sample size in our study may also have played a role. A study of arctic wolves in North America based on 14 microsatellites [37] observed a larger number of population clusters than a study of the same area (with fewer but more evenly distributed samples) using >26K SNP markers [61]. The higher mutation rate and variability in microsatellites may permit more rapid detection of population structure at very recent divergence times, although this could be balanced by employing a larger suite of SNP markers [62].

We identified certain canids with apparent dog ancestry, including four individuals from southern Ukraine where multiple instances of wolf-dog hybridization have been reported [63], [64]. However, the presence of these individuals did not alter the overall results. The putative hybrids appear to suggest the presence of back-crossed individuals (a first generation wolf-dog hybrid breeding back into the wolf population) in several European countries. Hybridization requires further investigation across Europe to determine the occurrence and extent of dog, and possible golden jackal, introgression, and how such processes may affect wolf genetic structure, behaviour, ecology, and interactions with humans ([6], [65], [66] and A.E. Moura, unpubl. data).

Within the Dinaric-Balkan cluster, we observed divergence between wolves from Croatia and individuals from Greece and Bulgaria. A recent evaluation of the European wolf distribution suggest relatively good landscape connectivity from the Carpathians and southward (Fig. 1), although the large Dinaric-Balkan wolf population likely exhibits substructuring [26]. Despite a bottleneck in the early 1990s, the present Croatian population appears to demonstrate high levels of genetic variation ([67] and references therein), and connectivity within Croatia has been well-preserved despite recent landscape development [68]. Croatian haplotypes have earlier been found to cluster with Bulgaria and the Alps [67]. Gene flow is expected to occur between the Croatian part of the Dinaric population and wolves in Slovenia, Bosnia & Herzegovina, and further southeast in Montenegro, Serbia and the former Yugoslav Republic of Macedonia [26], as well as with wolves in Bulgaria and Greece [67]. Previous investigations in Bulgaria and Greece also suggested relatively high haplotype diversity [28], [32], [69], and wolves from this part of the continent may encompass a significant portion of the diversity previously found in the large and continuous European population [28], [67]. Further sampling is needed to resolve the genetic structure in this part of Europe, and should aim to include samples from the area extending from Croatia southward to Bulgaria and Greece.

The definition of management units from population genetic data should consider the extent to which populations are demographically independent [70]. Such independence might be shaped, at least in part, by environmental and ecological influences on dispersal. Improved resolution of dispersal preferences could therefore inform conservation management in existing European populations and in areas presently being recolonized by wolves and other wide-ranging species.

## Supporting Information

Figure S1 **Principal component analysis of European wolves (n = 127) using 67K single nucleotide polymorphism (SNP) markers.** Genetic diversity is represented by distance and colour; individuals further away and with more different colours have more divergent genotypes. The first axis represents 3.6% of the variation, the second axis 2.4%.
(DOC)

Figure S2 **Principal component analysis of Italian wolves (n = 50) using 67K single nucleotide polymorphism (SNP) markers.** Genetic diversity is represented by distance and colour; individuals further away and with more different colours have more divergent genotypes. The first axis represents 5.7% of the variation, the second axis 4.3%.
(DOC)

Table S1 **Quality control of single nucleotide polymorphism (SNP) data from n = 272 canids (n = 96 Italian and n = 176 from other areas of Europe) for evaluation of wolf population structure.** The resulting data set had n = 177 samples (n = 50 from Italy and n = 127 from other areas of Europe).
(DOC)

Table S2 **Quality control of 79 462 single nucleotide polymorphism (SNP) loci in European wolf samples, resulting in a data set of 67 784 SNP loci.**
(DOC)

Table S3 **Summary of STRUCTURE results for Europe minus Italy and outliers (n = 127, 67K SNPs) for 3 repetitions of each K-value.** These suggest highest Delta K support for K2, then K4. (The very high value for K9 is not reliable as the runs for K10 did not converge).
(DOC)

Table S4 **Summary of STRUCTURE results for Italian wolves (n = 50, 67K SNPs) for 3 repetitions of each K-value.** The results suggest highest Delta K support for K2.
(DOC)
