# Supplementary figures and images for: North-South Differentiation and a Region of High Diversity in European Wolves (Canis lupus)
Source: PLoS One. 2013 Oct 11;8(10):e76454. doi: 10.1371/journal.pone.0076454 (PMC3795770; doi:10.1371/journal.pone.0076454)

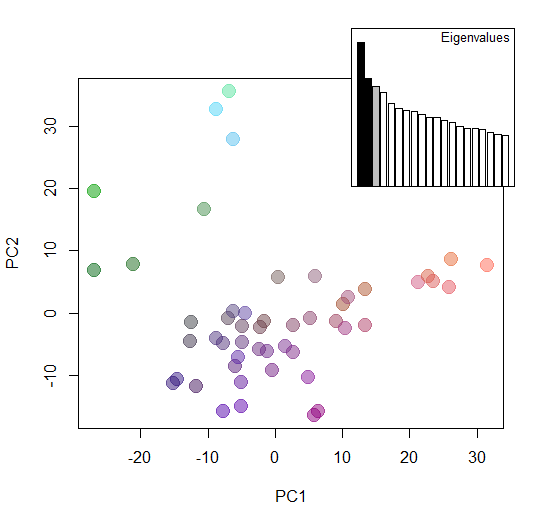

Supplement: Figure S2 — Principal component analysis of Italian wolves (n = 50) using 67K single nucleotide polymorphism (SNP) markers. Genetic diversity is represented by distance and colour; individuals further away and with more different colours have more divergent genotypes. The first axis represents 5.7% of the variation, the second axis 4.3%. (DOC) [file pone.0076454.s002.doc]
