# Supplementary material for: North-South Differentiation and a Region of High Diversity in European Wolves (Canis lupus)
Source: PLoS One. 2013 Oct 11;8(10):e76454. doi: 10.1371/journal.pone.0076454 (PMC3795770; doi:10.1371/journal.pone.0076454)
Supplement: Table S1 — Quality control of single nucleotide polymorphism (SNP) data from n = 272 canids (n = 96 Italian and n = 176 from other areas of Europe) for evaluation of wolf population structure. The resulting data set had n = 177 samples (n = 50 from Italy and n = 127 from other areas of Europe). (DOC) [file pone.0076454.s003.doc]

| **Criteria for removal** | **Italy** | **Europe other** | **Total** |
| --- | --- | --- | --- |
| Known dogs/hybrids | 23 | -- | 23 |
| Sample call rate < 60% | 9 | 9 | 18 |
| Relatedness > 0.5 | 13 | 34 | 47 |
| Extreme outliers^1^ | 1 | 6 | 7 |
| Total number of samples removed | 46 | 49 | 95 |

^1^Removed from final PCA and STRUCTURE analyses to improve resolution and representation of the remaining individuals.
