# Supplementary material for: North-South Differentiation and a Region of High Diversity in European Wolves (Canis lupus)
Source: PLoS One. 2013 Oct 11;8(10):e76454. doi: 10.1371/journal.pone.0076454 (PMC3795770; doi:10.1371/journal.pone.0076454)
Supplement: Table S2 — Quality control of 79 462 single nucleotide polymorphism (SNP) loci in European wolf samples, resulting in a data set of 67 784 SNP loci. (DOC) [file pone.0076454.s004.doc]

| **Criteria for removal** | **Number of SNP markers** |
| --- | --- |
| Linkage-disequilibrium pruning^1^ | 5378 |
| X-chromosome SNPs | 687 |
| Minor allele frequency (maf) < 0.01 | 23 |
| Genotyping rate (geno) < 0.02 | 5590 |
| Max individual missing rate (mind) = 0.4^2^ | 0 |
| Hardy Weinberg equilibrium (hwe) = 0^3^ | 0 |
| Total number of SNP loci removed | 11 678 |

^1^PLINK setting [--indep-pairwise 50 5 0.8]

^2^Corresponds to the removal of samples with sample call rate < 60% in Table S1, hence this screening had already been performed.

^3^Hardy-Weinberg equilibrium was not expected across Europe, and thus set to zero.
