# Supplementary material for: North-South Differentiation and a Region of High Diversity in European Wolves (Canis lupus)
Source: PLoS One. 2013 Oct 11;8(10):e76454. doi: 10.1371/journal.pone.0076454 (PMC3795770; doi:10.1371/journal.pone.0076454)
Supplement: Table S3 — Summary of STRUCTURE results for Europe minus Italy and outliers (n = 127, 67K SNPs) for 3 repetitions of each K-value. These suggest highest Delta K support for K2, then K4. (The very high value for K9 is not reliable as the runs for K10 did not converge). (DOC) [file pone.0076454.s005.doc]

| **K** | **Mean LnP(K)** | **Stdev LnP(K)** | **Ln'(K)** | **|Ln''(K)|** | **Delta K** |
| --- | --- | --- | --- | --- | --- |
| 1 | -7797902.63 | 508.61 | NA | NA | NA |
| 2 | -7673585.20 | 1085.49 | 124317.43 | 64030.00 | **58.99** |
| 3 | -7613297.77 | 3205.77 | 60287.43 | 9467.13 | 2.95 |
| 4 | -7562477.47 | 2334.17 | 50820.30 | 50760.53 | **21.75** |
| 5 | -7562417.70 | 57898.52 | 59.77 | 84562.30 | 1.46 |
| 6 | -7477795.63 | 9170.20 | 84622.07 | 152591.73 | 16.64 |
| 7 | -7545765.30 | 144062.10 | -67969.67 | 72834.77 | 0.51 |
| 8 | -7686569.73 | 461263.00 | -140804.43 | 338888.63 | 0.73 |
| 9 | -7488485.53 | 35640.51 | 198084.20 | 140840791.00 | 3951.71 |
| 10 | -148131192.33 | 53065296.10 | -140642706.80 | NA | NA |
