# Supplementary material for: North-South Differentiation and a Region of High Diversity in European Wolves (Canis lupus)
Source: PLoS One. 2013 Oct 11;8(10):e76454. doi: 10.1371/journal.pone.0076454 (PMC3795770; doi:10.1371/journal.pone.0076454)
Supplement: Table S4 — Summary of STRUCTURE results for Italian wolves (n = 50, 67K SNPs) for 3 repetitions of each K-value. The results suggest highest Delta K support for K2. (DOC) [file pone.0076454.s006.doc]

| **K** | **Mean LnP(K)** | **Stdev LnP(K)** | **Ln'(K)** | **|Ln''(K)|** | **Delta K** |
| --- | --- | --- | --- | --- | --- |
| 1 | -1831822.70 | 299.75 | NA | NA | NA |
| 2 | -1790940.07 | 708.88 | 40882.63 | 33782.03 | **47.66** |
| 3 | -1783839.47 | 12595.65 | 7100.60 | 178.77 | 0.01 |
| 4 | -1776560.10 | 8069.74 | 7279.37 | 27377.73 | 3.39 |
| 5 | -1796658.47 | 24870.11 | -20098.37 | 33190.33 | 1.33 |
| 6 | -1783566.50 | 15775.02 | 13091.97 | 13199.77 | 0.84 |
| 7 | -1783674.30 | 13172.95 | -107.80 | 61542.10 | 4.67 |
| 8 | -1845324.20 | 102479.05 | -61649.90 | 113665.50 | 1.11 |
| 9 | -1793308.60 | 22986.58 | 52015.60 | 39177.60 | 1.70 |
| 10 | -1780470.60 | 10180.34 | 12838.00 | NA | NA |
